# Perceived racial discrimination, childhood adversity, and self-reported high blood pressure among adults in rural Alabama

**DOI:** 10.3389/fpubh.2025.1575793

**Published:** 2025-07-11

**Authors:** Tenesha Littleton, Luciana Giorgio Cosenzo, Joana Okine, Sharlene D. Newman

**Affiliations:** ^1^School of Social Work, The University of Alabama, Tuscaloosa, AL, United States; ^2^Alabama Life Research Institute, The University of Alabama, Tuscaloosa, AL, United States

**Keywords:** hypertension, racial discrimination, childhood adversity, trauma, rural populations, health disparities

## Abstract

**Introduction:**

Disproportionate exposure to psychosocial stressors, such as racial discrimination, and other forms of adversity across the life course contributes to higher rates of hypertension among Black Americans. However, prior literature is limited by the underrepresentation of rural populations and narrow measurements of racial discrimination. This study examines associations between perceived racial discrimination (through a historical trauma lens), childhood adversity, and self-reported high blood pressure among adults living in predominantly Black communities in rural Alabama.

**Methods:**

Data were collected using paper-and-pencil surveys from 184 participants across five rural communities in Alabama in Spring 2023. High blood pressure was assessed via self-report from a list of chronic conditions. Perceived racial discrimination was measured by a 5-item subscale of the African American Historical Trauma Questionnaire. Childhood adversity was measured by the 10-item ACE Study Questionnaire. Binary logistic regression examined associations between high blood pressure, childhood adversity, and perceived racial discrimination, adjusting for psychological distress and sociodemographic factors.

**Results:**

Ninety-two percent of the sample were Black Americans. Older age (OR = 1.09, 95% CI = 1.05, 1.12) and higher perceived racial discrimination scores (OR = 1.15, 95% CI = 1.04, 1.27) were significantly associated with increased odds of high blood pressure.

**Discussion:**

Findings highlight the importance of multilevel interventions that are both trauma-informed and culturally tailored to reduce health disparities in rural Black communities.

## Introduction

Hypertension (also referred to as high blood pressure herein) is a public health epidemic both nationally and globally. In the United States (US), approximately half of adults (48.1%) have been diagnosed with hypertension based on the new American College of Cardiology and the American Heart Association defining hypertension as a blood pressure of 130/80 mmHg or above (1, 2). The prevalence of hypertension is highest in rural areas of the US and the Deep South (3, 4). Hypertension rates for the state of Alabama are among the highest in the country (4) where 82% of the counties in the state are classified as rural areas (5). Hypertension has consistently been associated with an increased risk of cardiovascular disease, stroke, and premature death (1, 6, 7). Additionally, individuals with hypertension, on average, experience $2000 more in healthcare expenditures than their non-hypertensive counterparts (8). It has been estimated that hypertension accounts for $131 billion annually in the US (8).

In the US, racial disparities exist in the prevalence of hypertension with Black Americans experiencing a significantly higher prevalence of this condition than White Americans (56% vs. 48%, respectively) (1, 9, 10). Alarmingly, Black Americans have an earlier onset of hypertension and experience greater complications from this condition than their White counterparts (11). The reasons for this disparity are complex however, research suggests that disproportionate exposure to psychosocial stressors, such as racial discrimination, and adversity across the life course contributes to the higher rates of hypertension among Black Americans (12). Chronic exposure to psychosocial stressors can trigger a cascade of physiological responses across multiple systems within the body that regulate stress including the nervous, neuroendocrine, and immune systems (13, 14). When experiencing stress, blood pressure rises in response to the release of hormones (i.e., adrenaline and cortisol) which increase heart rate and narrow the blood vessels to prepare the body to respond to the stressor (15). Repeated activation of this biological stress response can result in failing to return to resting blood pressure levels (12).

Structural racism has resulted in unequal access to the social determinants of health for Black Americans, exposing them to a higher burden of stress than White Americans (16). For example, due to a long history of discriminatory policies that have limited access to economic mobility and intergenerational wealth transfers, Black Americans earn less and have less wealth than White Americans (17). Multiple indicators of socioeconomic status have been linked to hypertension (18). The racial wealth gap limits access to occupational, educational, and social resources that promote health while also limiting the ability to cope with the accumulation of chronic stressors that increase the risk of hypertension (12). In addition, because of persistent racial residential segregation, Black Americans are more likely to reside in high poverty neighborhoods with less green space (19), reduced access to healthy food options (20), and higher crime rates (21). These social determinants of health impact health disparities directly (22) and indirectly by increasing the risk of experiencing adverse childhood experiences (ACEs) (23).

ACEs are traumatic events that occur during the first 18 years of life and include physical, sexual, and emotional abuse, neglect, as well as experiences of household dysfunction such as parental separation or divorce, witnessing intimate partner violence against the mother, and living with an adult experiencing mental illness, substance abuse, or incarceration (24). ACEs are common among adults in the US however, Black Americans experience a greater number of ACEs compared to other racial and ethnic groups (25, 26). A dose-effect relationship has been observed between ACEs and negative outcomes including higher risk of mental and physical health problems (27). Research examining the link between ACEs and hypertension have been limited and findings have produced mixed results. A study analyzing data spanning 10 countries from the World Mental Health Survey found that two or more ACEs was associated with self-reported hypertension (28). Similarly, a study of 12,229 low-income adults in the US also found an association between ACEs and self-reported history of hypertension (29). However, other studies did not observe a relationship (30, 31). The mixed findings have been attributed to inconsistences in how childhood adversity and blood pressure are measured (32).

The traditional ACEs framework has been critiqued for not including racial discrimination as a traumatic event (33). Racial discrimination refers to unfair treatment based on racial identity. It can take various forms including interpersonal interactions or institutional practices that systematically treat certain groups unfavorably (34). Racial discrimination is often perceived as a threat to safety that can trigger intense emotional reactions and possibly lead to race-based traumatic stress (35). Race-based traumatic stress can mimic reactions observed among individuals experiencing Post-Traumatic Stress Disorder including avoidance, hypervigilance, and severe psychological distress (35). Racial discrimination is a distinct type of ACE that also increases the risk for other ACEs and restricts access to the supportive resources needed to help buffer against adverse physical and mental health outcomes (33).

Acts of racial discrimination do not have to be directly experienced to induce race-based traumatic stress for group members. Traumatic events include those that are observed or vicariously experienced (36). Theories of historical trauma suggests that mass level collective experiences of racism across multiple generations can contribute to the adverse physical and mental health functioning of future generations (37). For Black Americans, historical trauma is “the collective spiritual, psychological, emotional, and cognitive distress perpetuated intergenerationally deriving from multiple denigrating experiences originating with slavery and continuing with pattern forms of racism and discrimination to the present day” (p. 32) (38). Historical trauma is transmitted intergenerationally through biological, epigenetic, environmental, social, economic, and political systems (39, 40). In addition, technological advances and the proliferation of social media increases widespread exposure to traumatic events that can lead to vicarious encounters of racism and symptoms of traumatic stress (41).

Evidence of a positive association between racial discrimination and hypertension, specifically among Black Americans, has been increasing over the past two decades (42–44). Although studies have yielded mixed results, most findings suggest racial discrimination partially explains the racial disparities in the prevalence of hypertension seen among Black vs. White Americans. The variance in study results has been attributed to inconsistencies in the measurement of racial discrimination (e.g., everyday discrimination, lifetime discrimination) (42). Results from studies using a lifetime measurement of discrimination have demonstrated more consistently a significant and positive association between racial discrimination and hypertension among Black Americans. For example, a recent study using longitudinal data from the Jackson Heart Study, found that medium to high reports of lifetime racial discrimination were significantly associated with a higher risk of hypertension compared to low lifetime racial discrimination, even when adjusting for hypertension risk factors (45). However, the association between everyday discrimination and hypertension risk was not statistically significant (45). Yet even the lifetime discrimination scale is limited to capturing daily experiences at interpersonal and institutional levels while failing to capture the collective experiences of racial trauma that impact the psyche of Black Americans that a historical trauma framework suggests (46). Other measures of racial discrimination, such as the African American Historical Trauma Questionnaire, can capture perceptions of discrimination originating from the enduring effects of slavery, structural racism, and other traumatic events Black Americans have experienced and that are passed down intergenerationally (46). To date, the association between hypertension and racial discrimination from a historical trauma perspective has not been examined.

The purpose of this study is to examine the association between childhood adversity, perceived racial discrimination, and self-reported high blood pressure among a sample of adults living in predominantly Black communities in rural Alabama. This study adds to the literature in three important ways. First, we use a novel measure of perceived racial discrimination from a historical trauma perspective rather than focusing on direct, everyday experiences of racial discrimination that are typical of research in this area. Second, this study focuses on an underserved, rural population that is often underrepresented in research studies of hypertension (47). This is particularly troubling considering that for the last two decades, mortality rates for hypertension have consistently been highest among Black adults living in rural parts of the United States (48). Lastly, our study controls for childhood adversity and psychological distress which can confound the association between perceived racial discrimination and hypertension and are often not included as variables in prior research.

## Materials and methods

### Study population and design

The data collection for this cross-sectional study was a part of an ongoing research collaboration between the University and five rural, predominately Black communities in the Alabama Black Belt Region. Originally named for its fertile soil, the Black Belt region has a long history of racial and economic injustice dating back to chattel slavery and the Jim Crow era. The region is home to some of the poorest counties in the country and experiences the worst health outcomes in Alabama (49, 50). The goal of the research collaboration is to use community-based participatory approaches to improve the health outcomes of these communities [for a more detailed discussion of the community-academic partnership see Newman et al. (51)]. Using a convenience sampling strategy, community representatives recruited participants at community events, local stores, government offices, and churches. One hundred and eighty-four participants completed the 34-item survey at community sites in the Spring of 2023. This study was approved by the Institutional Review Board of the University of Alabama.

### Measures

#### Dependent variable

Participants reported whether they had high blood pressure by selecting from a list of chronic health conditions. High blood pressure was measured as a dichotomized variable (0 = no, 1 = yes).

#### Sociodemographic characteristics

Age, gender, education, and marital status were included as covariates in this study. Age was measured in years as a continuous variable. Participants were asked, “How many years of school have you completed?” and response options included: less than high school, high school graduate, some college, associate degree, bachelor’s degree, and graduate degree or higher. The variable was recoded into a dichotomous variable due to low observations in multiple categories (0 = high school graduate or less, 1 = some college or higher). Gender (0 = male, 1 = female) and marital status (0 = not married, 1 = married) were also analyzed as dichotomous variables.

#### Childhood adversity

Childhood adversity was measured by the ACE Study Questionnaire (24). This 10-item questionnaire asks participants to indicate (yes/no) if they had experienced abuse or other household dysfunction during the first 18 years of life. The categories of abuse include emotional abuse, emotional neglect, physical neglect, physical abuse, and sexual abuse. The categories of household dysfunction include exposure to substance use, mental illness, intimate partner violence against the mother or stepmother, and the incarceration of a household member. An ACE score was derived for each participant by summing the number of yes responses to the questions.

#### Perceived racial discrimination

Perceived racial discrimination was measured by a subscale of the African American Historical Trauma Questionnaire (46). Using a 4-point Likert scale (ranging from 0 = never to 3 = always), this 5-item measure asks participants to indicate: (1) “How often do you believe life is an uphill battle?” (2) “How often do you believe you are constantly being held back?” (3) “How often do you believe African Americans typically endure unnecessary hardship due to race?” (4) “How often do you believe the world is against you just because you are African American?” (5) “How often do you believe it is necessary to work twice as hard to succeed as an African American?” Responses to the items were summed to create a perceived racial discrimination score ranging from 0 to 15 (Cronbach’s alpha = 0.92).

#### Psychological distress

Psychological distress was assessed using the Patient Health Questionnaire for Depression and Anxiety (PHQ-4) (52). This 4-item measure assesses symptoms of depression and anxiety (e.g., feeling nervous on edge, feeling down or hopeless) using a 4-point Likert scale with response options ranging from 0 = never to 3 = nearly every day. Responses to the items were summed to create a psychological distress score ranging from 0 to 12 (Cronbach’s alpha = 0.93).

### Analytic strategy

Before conducting analyses, data were screened for missing values. Percent missing among the variables ranged from 0 to 14.1%. Variables with more than 10% missing were recoded with missing values coded as 1 and all other values coded as 0. Bivariate analyses were performed between the recoded variables and all other variables to determine if relationships were present. Effect sizes did not exceed 0.5, indicating that no patterns were present, and the data were missing at random. Thus, the pairwise deletion method was applied for missing data.

Descriptive analyses for all variables were conducted to analyze the characteristics of the sample. Bivariate analyses were conducted to assess the relationships between self-reported high blood pressure and independent variables using independent sample t-test for continuous independent variables and chi squares for dichotomous independent variables. Binary logistic regression was used to examine the relationship between self-reported high blood pressure, childhood adversity, and perceived racial discrimination while controlling for psychological distress and sociodemographic characteristics. Data analyses were performed using IBM SPSS Version 27.

## Results

### Descriptive and bivariate analyses

**Figure 1:**
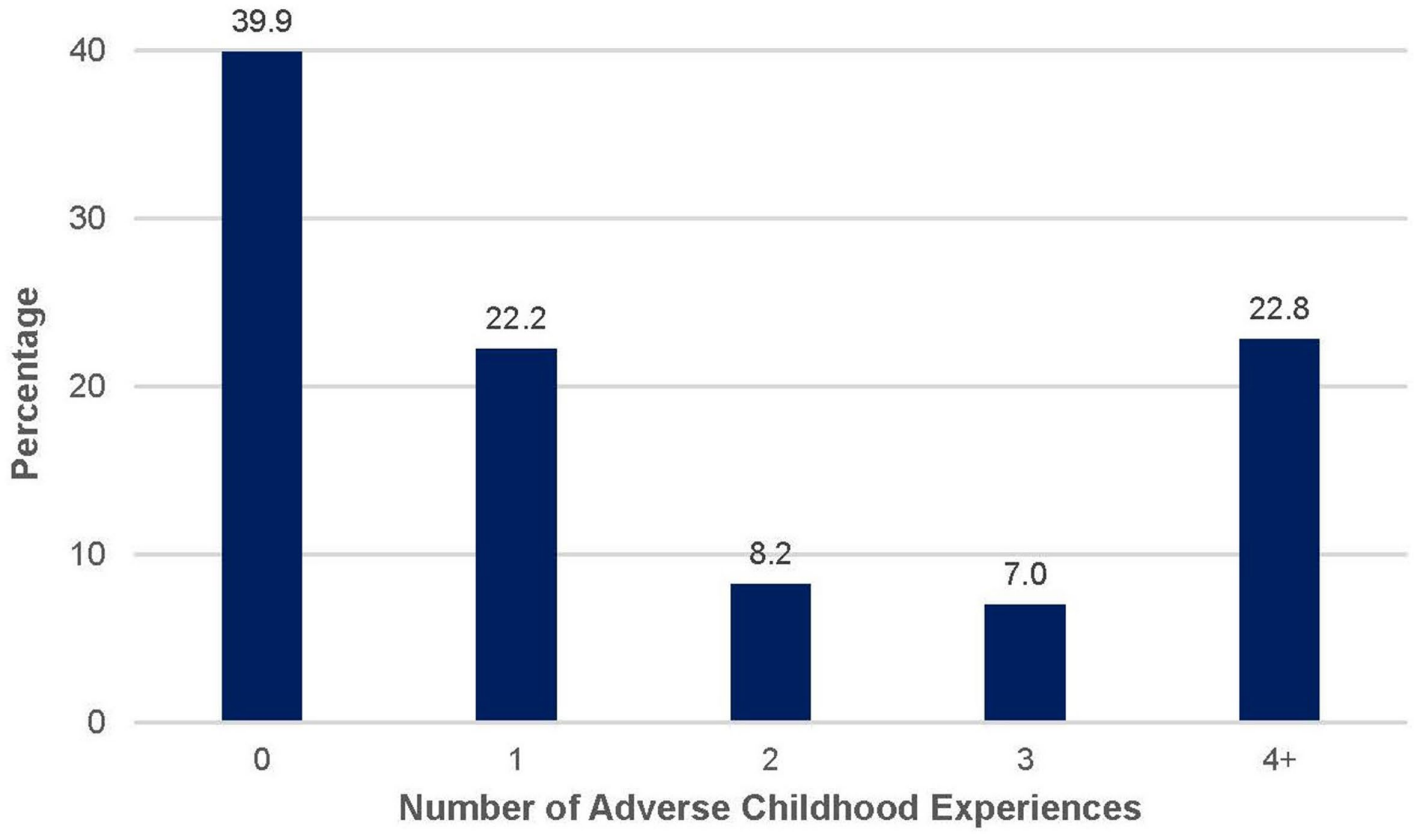
Total number of adverse childhood experiences reported by participants. Bar graph displaying the percentage of participants (*N* = 184) by total ACE score. Percentages are based on valid responses; sample size may vary slightly across items due to missing data.

**Figure 2:**
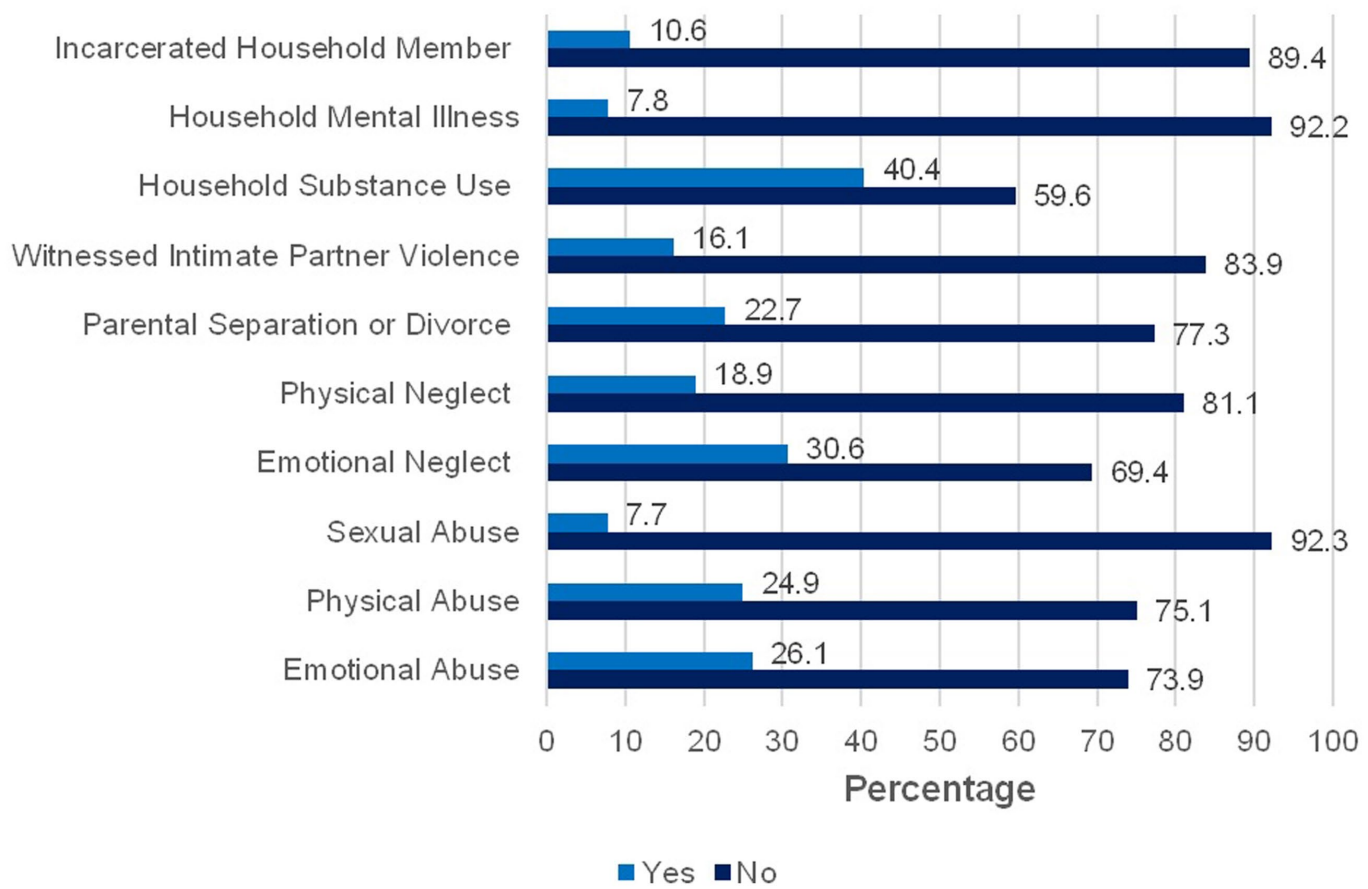
Prevalence of each adverse childhood experience in the study sample. Bar chart displaying the percentage of participants (*N* = 184) who reported experiencing each type of adverse childhood experience. Percentages are based on valid responses; sample size may vary slightly across items due to missing data.

Descriptive and bivariate analyses are presented in Table 1. About 63% of the sample reported high blood pressure. The mean ACE score was 1.89 (SD = 2.32). Figure 1 displays the percentage of participants by total ACE score. Figure 2 illustrates the prevalence of each type of ACE reported by participants. The mean discrimination score was 8.81 (SD = 4.76). The mean psychological distress score was 1.7 (SD = 2.60) with about 10% of the sample experiencing moderate to severe psychological distress. The mean age was 50.63 (SD = 18.58). About 92% of the sample was Black/African American. Most participants were women (57.6%), unmarried (51.6%), and did not have any college experience (79.6%).

**Table 1:** Descriptive characteristics by high blood pressure status.

Variable	All	No HBP	Yes HBP
No. (*N*)	184	69	115
Proportion of sample (%)	100%	37.5%	62.5%
Age, mean (SD)	50.6 (18.58)	40.50 (17.10)***	56.78 (16.71)
Sex, *n* (%)			
Male	71(40.1)	30 (42.3)	41 (57.7)
Female	106 (59.9)	36 (34.0)	70 (66.0)
Marital status, *n* (%)			
Married	70 (42.4)	25 (35.7)	45 (64.3)
Not Married	95 (57.6)	35 (36.8)	60 (63.2)
Education, *n* (%)			
<High School Graduate	144 (81.8)	55 (37.8)	89 (78.8)
≥Some College	37 (18.2)	13 (36.4)	24 (64.9)
Race, *n* (%)			
Black	167 (92.3)	60 (35.9)	107 (64.1)
White	14 (7.6)	8 (57.1)	6 (42.9)
ACE Score, mean (SD)	1.88 (2.32)	1.69 (2.42)	2.01 (2.25)
Psychological Distress, mean (SD)	1.70 (2.60)	1.30 (2.64)	1.94 (2.56)
Discrimination Score, mean (SD)	8.81 (4.76)	7.02 (4.93)***	9.92 (4.76)

***p < 0.001.

At the bivariate level, participants with high blood pressure endorsed higher levels of perceived racial discrimination, *t*(170) = −4.074, *p* = 0.000. Participants with high blood pressure were also older on average than participants who did not report high blood pressure, *t*(178) = −6.282, *p* = 0.000. The other variables were not associated with high blood pressure.

### Binary logistic regression

The results of the binary logistic regression are presented in Table 2. Older age (*OR* = 1.09, 95% CI = 1.05, 1.13) and higher perceived racial discrimination scores were associated with higher odds of high blood pressure (*OR* = 1.15, 95% CI = 1.04, 1.27). ACE score was not associated with high blood pressure. All variance inflation factor (VIF) values were close to 1, indicating that multicollinearity was not a concern. The model demonstrated good fit (Hosmer–Lemeshow *χ*^2^ = 10.25, *p* = 0.25) and strong discriminatory ability (AUC = 0.847), indicating high accuracy in distinguishing individuals with and without self-reported high blood pressure. The model also explained 44.7% of the variance in the dependent variable (Nagelkerke *R*^2^ = 0.447).

**Table 2:** Logistic regression analysis of childhood adversity, perceived racial discrimination, and high blood pressure status (*N* = 184^a^).

Variable	OR for high blood pressure status
	OR (95% CI)
ACE score	1.09 (0.87, 1.36)
Perceived discrimination	1.15 (1.04, 1.27)**
Age, y	1.09 (1.05, 1.12)***
Marital Status	
Not Married	Reference
Married	1.54 (0.59, 4.00)
Sex	
Male	Reference
Female	0.394 (0.16, 1.00)
Educational Attainment	
<High School Graduate	Reference
≥Some College	1.67 (0.57, 4.91)
Psychological distress	0.96 (0.78, 1.18)
Observations	126^a^
Pseudo *R*-square	0.447

^a^The total sample size of the regression may not be the same as the total sample size of the study due to missing values. OR = Odds Ratio; 95% CI = 95% Confidence Interval.

**p < 0.01, ***p < 0.001.

## Discussion

This study examined the relationship between perceived racial discrimination, childhood adversity, and self-reported high blood pressure among a sample of adults from rural, majority Black communities in Alabama. This study sought to address important gaps in the literature by examining high blood pressure risk among adults in under-resourced communities in the rural Deep South utilizing a historical trauma lens to conceptualize perceived racial discrimination while controlling for psychological distress and childhood adversity. Older age and higher levels of perceived discrimination were associated with higher odds of high blood pressure.

The prevalence of high blood pressure in the sample was seven percentage points above the national average for Black Americans (3). The higher rates are likely attributed to characteristics of the sample and the study location. The Deep South has consistently had the highest prevalence of hypertension in the United States with Alabama having rates among the highest in the country (3). Second, the sample overall is older, with half of participants at age 50 or older. Rates of chronic diseases including high blood pressure tend to increase with age (53) which is consistent with our finding that higher age was associated with increased odds of high blood pressure.

High levels of perceived racial discrimination were present among participants in the sample. The legacy of chattel slavery and Jim Crow still shapes the social and economic context of communities in the Deep South negatively impacting intergenerational mobility in the region (54, 55). Rather than focus on direct, individual experiences of discrimination, our broad measure captures generalized perceptions of disparate treatment and lower quality of life because of racial identity that aligns with a historical trauma perspective. Consistent with some prior research using conventional measures of racial discrimination, higher levels of perceived racial discrimination were associated with increased odds of high blood pressure in our study. For example, everyday racial discrimination was associated with increased odds of self-reported hypertension among Black women with higher levels of education (56). Similarly, a longitudinal study of a multi-ethnic cohort of 3,297 adults found that lifetime discrimination was associated with incident hypertension among Black Americans in the sample (57). Research suggests that two possible pathways link perceived racial discrimination to increased risk of high blood pressure. First, race-based traumatic stress can trigger repeated activation of the stress response leading to physiological changes in the body, including difficulty returning to resting levels of blood pressure. The weathering hypothesis suggests that chronic stress and other accumulated disadvantages over the life course can lead to premature biological aging and health disparities among Black Americans (58, 59). Second, the psychological impact of perceived racial discrimination can contribute to difficulty engaging in health promoting behaviors due to unhealthy coping strategies (60).

ACEs were also common among participants. Sixty percent of the sample experienced at least one ACE which is fairly consistent with national estimates (61). About a quarter of the sample experienced four or more ACEs which is higher than national estimates indicating that 17% of the general population has experienced four or more ACEs (61). The greater likelihood of experiencing multiple ACEs in the sample is consistent with prior research that has found a higher prevalence of ACEs among Black Americans (25, 26). These disparities in ACEs prevalence have been attributed to the inequitable social and economic contexts that Black families disproportionately experience.

Consistent with some prior research, the number of ACEs was not associated with high blood pressure. For example, a population level cohort study of US middle aged adults did not find a direct association between ACEs and self-reported hypertension (30). Similarly, a study of childhood adversity and hypertension among mostly midlife women found no direct association (31). Like our study, these studies included mostly middle aged and older adults, which may partially explain the null findings. Research suggests that developmental timing may be important for detecting differences in risk of high blood pressure related to ACEs. For example, Su et al. (62) examined the long-term effect of ACEs on blood pressure trajectories from childhood to young adulthood and found a statistically significant association between the number of ACEs and longitudinal blood pressure trajectories such that the effect sizes increased as the participants aged into young adulthood. Similarly, among a sample of 45,482 participants ranging in age from 18 to 99 years, a dose effect of ACEs on hypertension risk was most pronounced among the younger adults (63). Thus, the risk associated with developing high blood pressure may be elevated at earlier ages for those with a history of ACEs compared to those without a history (62). In addition, as noted previously, the traditional ACEs framework does not include racial discrimination which may lead to an underestimation of childhood adversity within this sample and contribute to the null findings. Moreover, the traditional ACEs framework focuses solely on household-level dysfunction and fails to account for the community-level adversities (e.g., concentrated poverty, failing schools) that can impact health and wellbeing over time. This limitation is particularly relevant for the Black Belt region where communities face significant structural inequities.

### Limitations and directions for future research

This exploratory study adds to the relatively scarce literature examining factors that may be associated with high blood pressure among Black Americans in rural, under-resourced communities, however there are several noteworthy limitations. First, this study is cross-sectional. Thus, causation and the temporal direction of the relationships observed in this study cannot be ascertained. Future studies should incorporate longitudinal research designs with larger samples from these communities. Second, the sample was convenient and thus not representative of the population, limiting the generalizability of our findings. Third, our measure of high blood pressure is self-reported as clinical measures of blood pressure were not collected. While self-reported high blood pressure has demonstrated reasonable concordance with clinical measurements in large scale epidemiological studies (64), it may introduce recall bias or risk of misclassification. Similarly, our study did not collect other biomarker data such as body mass index, or other hypertension risk factors like smoking status or physical activity. However, it is important to note that prior research has found a statistically significant relationship between racial discrimination and hypertension when controlling for these risk factors (42, 54). Fourth, psychological distress was included as a control variable to account for potential confounding. However, as a possible mediator between perceived racial discrimination and high blood pressure, its inclusion could introduce overadjustment bias and attenuate observed associations. Lastly, our use of the African American Historical Trauma Questionnaire is exploratory, and additional research is needed to examine its association with health outcomes among Black Americans.

### Implications for public health interventions

Public health efforts to address health disparities in rural Black communities should utilize multilevel strategies that include community-based participatory approaches to engage community members in identifying community needs and developing culturally relevant solutions. First, it is critical to address the social drivers of health and rectify systemic inequities in health care infrastructure, housing, and transportation through targeted policy investments in rural Black communities. These systemic barriers make it difficult for people to adopt the lifestyle modifications that help reduce risk of hypertension. At the micro level, trauma-informed primary care (TIPC) models should be implemented in rural Black communities. TIPC involves screening for patient trauma history (including race-based traumatic stress), educating patients about the effect of stress on health, emphasizing cultural strengths in managing health, and making appropriate referrals for mental health care (65). In addition, programs like Mental Health First Aid teaches laypersons in the community how to identify, understand, and respond to signs of mental illness and substance abuse (66). MHA has shown promise in raising awareness of behavioral health issues and reducing stigma in rural communities (66). MHA also aligns with the tradition of mutual aid among Black Americans and the strong informal networks of support present in many rural communities (67). Lastly, public health interventions should include efforts to help communities heal from racial trauma through the use of expressive arts such as storytelling to create counternarratives that emphasize collective strength and affirm the dignity and worth of Black identity (39, 68). The reframing of harmful narratives can help empower Black communities to increase individual agency and spur collective social action to improve the health and wellbeing of the community.

## Data Availability

The raw data supporting the conclusions of this article will be made available by the authors, without undue reservation.

## References

[1] CDC, “Facts about hypertension | cdc.Gov,” Ctr Dis Control Prev. Available online at: https://www.cdc.gov/bloodpressure/facts.htm (accessed October 03, 2023)

[2] Whelton PK Carey RM Aronow WS Casey DE Collins KJ Dennison Himmelfarb C. 2017 ACC/AHA/AAPA/ABC/ACPM/AGS/APhA/ASH/ASPC/NMA/PCNA guideline for the prevention, detection, evaluation, and management of high blood pressure in adults: executive summary: a report of the American College of Cardiology/American Heart Association task force on clinical practice guidelines. Hypertension. (2018) 71:1269–324. doi: 10.1161/HYP.0000000000000066, PMID: 29133354

[3] CDC, “Facts about hypertension | cdc.Gov,” Centers for Disease Control and Prevention. Available online at: https://www.cdc.gov/bloodpressure/facts.htm (accessed September 01, 2023)

[4] Explore high blood pressure in Alabama | AHR. America’s health rankings. Available online at: https://www.americashealthrankings.org/explore/measures/hypertension/AL (accessed: July 27, 2023)

[5] Analysis of Urban vs. rural, Alabama rural health association. Available online at: https://arhaonline.org/analysis-of-urban-vs-rural/ (accessed March 29, 2024)

[6] Chobanian AV Bakris GL Black HR Cushman WC Green LA Izzo JL. The seventh report of the joint National Committee on prevention, detection, evaluation, and treatment of high blood pressure: the JNC 7 report. JAMA. (2003) 289:2560–72. doi: 10.1001/jama.289.19.2560, PMID: 12748199

[7] O’donnell MJ Xavier D Liu L Zhang H Chin SL Rao-Melacini P. Risk factors for ischaemic and intracerebral haemorrhagic stroke in 22 countries (the INTERSTROKE study): a case-control study. Lancet. (2010) 376:112–23. doi: 10.1016/S0140-6736(10)60834-3 20561675

[8] Kirkland EB Heincelman M Bishu KG Schumann SO Schreiner A Axon RN. Trends in healthcare expenditures among US adults with hypertension: National Estimates, 2003–2014. J Am Heart Assoc. (2018) 7:e008731. doi: 10.1161/JAHA.118.008731, PMID: 29848493 PMC6015342

[9] Dorans KS Mills KT Liu Y He J. Trends in prevalence and control of hypertension according to the 2017 American College of Cardiology/American Heart Association (ACC/AHA) guideline. J Am Heart Assoc. (2018) 7:e008888. doi: 10.1161/JAHA.118.008888, PMID: 29858369 PMC6015372

[10] Ogunniyi MO Commodore MY Ferdinand KC. Race, ethnicity, hypertension, and heart disease. J Am Coll Cardiol. (2021) 78:2460–70. doi: 10.1016/j.jacc.2021.06.017 34886968

[11] Ferdinand KC Townsend RR. Hypertension in the US Black population: risk factors, complications, and potential impact of central aortic pressure on effective treatment. Cardiovasc Drugs Ther. (2012) 26:157–65. doi: 10.1007/s10557-011-6367-8, PMID: 22246101

[12] Cuevas AG Williams DR Albert MA. Psychosocial factors and hypertension: a review of the literature. Cardiol Clin. (2017) 35:223–30. doi: 10.1016/j.ccl.2016.12.004, PMID: 28411896 PMC5407387

[13] Black PH Garbutt LD. Stress, inflammation and cardiovascular disease. J Psychosom Res. (2002) 52:1–23. doi: 10.1016/S0022-3999(01)00302-6, PMID: 11801260

[14] Spruill TM. Chronic psychosocial stress and hypertension. Current Science Inc. (2010) 12:10–6. doi: 10.1007/s11906-009-0084-8, PMID: 20425153 PMC3694268

[15] Curtis BM O’Keefe JH. Autonomic tone as a cardiovascular risk factor: the dangers of chronic fight or flight. Mayo Clin Proc. (2002) 77:45–54. doi: 10.4065/77.1.45 11794458

[16] Myers HF. Ethnicity- and socio-economic status-related stresses in context: an integrative review and conceptual model. J Behav Med. (2009) 32:9–19. doi: 10.1007/s10865-008-9181-4, PMID: 18989769

[17] Aladangady A. Forde A. Wealth inequality and the racial wealth gap. (2021). Available online at: https://www.federalreserve.gov/econres/notes/feds-notes/wealth-inequality-and-the-racial-wealth-gap-20211022.html (accessed January 30, 2025)

[18] Leng B Jin Y Li G Chen L Jin N. Socioeconomic status and hypertension: a meta-analysis. J Hypertens. (2015) 33:221. doi: 10.1097/HJH.0000000000000428, PMID: 25479029

[19] Casey JA James P Cushing L Jesdale BM Morello-Frosch R. Race, ethnicity, income concentration and 10-year change in urban greenness in the United States. Int J Environ Res Public Health. (2017) 14:1546. doi: 10.3390/ijerph14121546, PMID: 29232867 PMC5750964

[20] Bower KM Thorpe RJ Rohde C Gaskin DJ. The intersection of neighborhood racial segregation, poverty, and urbanicity and its impact on food store availability in the United States. Prev Med. (2014) 58:33–9. doi: 10.1016/j.ypmed.2013.10.010, PMID: 24161713 PMC3970577

[21] Krivo LJ Peterson RD Kuhl DC. Segregation, racial structure, and neighborhood violent crime. Am J Sociol. (2009) 114:1765–802. doi: 10.1086/597285, PMID: 19852253

[22] Kramer MR Hogue CR. Is segregation bad for your health? Epidemiol Rev. (2009) 31:178–94. doi: 10.1093/epirev/mxp001, PMID: 19465747 PMC4362512

[23] Maguire-Jack K Font S Dillard R Dvalishvili D Barnhart S. Neighborhood poverty and adverse childhood experiences over the first 15 years of life. Int Journal on Child Malt. (2021) 4, 93–114. doi: 10.1007/s42448-021-00072-y

[24] Felitti VJ Anda RF Nordenberg D Williamson DF Spitz AM Edwards V. Relationship of childhood abuse and household dysfunction to many of the leading causes of death in adults: the adverse childhood experiences (ACE) study. Am J Prev Med. (1998) 14:245–58. doi: 10.1016/S0749-3797(98)00017-8 9635069

[25] Slopen N Shonkoff JP Albert MA Yoshikawa H Jacobs A Stoltz R. Racial disparities in child adversity in the U.S. Am J Prev Med. (2016) 50:47–56. doi: 10.1016/j.amepre.2015.06.013, PMID: 26342634

[26] Merrick MT Ford DC Ports KA Guinn AS. Prevalence of adverse childhood experiences from the 2011-2014 behavioral risk factor surveillance system in 23 states. JAMA Pediatr. (2018) 172:1038–44. doi: 10.1001/jamapediatrics.2018.2537, PMID: 30242348 PMC6248156

[27] Petruccelli K Davis J Berman T. Adverse childhood experiences and associated health outcomes: a systematic review and meta-analysis. Child Abuse Negl. (2019) 97:104127. doi: 10.1016/j.chiabu.2019.104127, PMID: 31454589

[28] Stein DJ Scott K Haro Abad JM Aguilar-Gaxiola S Alonso J Angermeyer M. Early childhood adversity and later hypertension: data from the world mental health survey. Ann Clin Psychiatry. (2010) 22:19–28. doi: 10.1177/104012371002200104 20196979 PMC3486699

[29] Allen H Wright BJ Vartanian K Dulacki K Li H-F. Examining the prevalence of adverse childhood experiences and associated cardiovascular disease risk factors among low-income uninsured adults. Circ Cardiovasc Qual Outcomes. (2019) 12:e004391. doi: 10.1161/CIRCOUTCOMES.117.004391, PMID: 31450964

[30] Matthews TA Zhu Y Robbins W Rezk-Hanna M Macey PM Song Y. Adulthood psychosocial disadvantages and risk of hypertension in U.S. workers: effect modification by adverse childhood experiences. Life. (2022) 12:1507. doi: 10.3390/life12101507, PMID: 36294941 PMC9604677

[31] Cubbin C Kim Y Panisch LS. Familial childhood adversity is associated with chronic disease among women: data from the geographic research on wellbeing (GROW) study. Matern Child Health J. (2019) 23:1117–29. doi: 10.1007/s10995-019-02758-9, PMID: 31203522 PMC7727326

[32] Scott J McMillian-Bohler J Johnson R Simmons LA. Adverse childhood experiences and blood pressure in women in the United States: a systematic review. J Midwifery Womens Health. (2021) 66:78–87. doi: 10.1111/jmwh.13213, PMID: 33576175 PMC8170683

[33] Bernard DL Calhoun CD Banks DE Halliday CA Hughes-Halbert C Danielson CK. Making the ‘C-ACE’ for a culturally-informed adverse childhood experiences framework to understand the pervasive mental health impact of racism on Black youth. J Child Adolesc Trauma. (2020) 14:233–47. doi: 10.1007/s40653-020-00319-9, PMID: 33986909 PMC8099967

[34] Lockwood KG Marsland AL Matthews KA Gianaros PJ. Perceived discrimination and cardiovascular health disparities: a multisystem review and health neuroscience perspective. Ann N Y Acad Sci. (2018) 1428:170–207. doi: 10.1111/nyas.13939, PMID: 30088665

[35] Carter RT Mazzula S Victoria R Vazquez R Hall S Smith S. Initial development of the race-based traumatic stress symptom scale: assessing the emotional impact of racism. Psychol Trauma Theory Res Pract Policy. (2013) 5:1–9. doi: 10.1037/a0025911

[36] American Psychiatric Association. Diagnostic and statistical manual of mental disorders. 5th ed. Arlington (VA): American Psychiatric Publishing (2013).

[37] Heart MYHB. The historical trauma response among natives and its relationship with substance abuse: a Lakota illustration. J Psychoactive Drugs. (2003) 35:7–13. doi: 10.1080/02791072.2003.10399988, PMID: 12733753

[38] Hampton RL Gullotta TP Crowel RL. Handbook of African American health In: Handbook of African American health. New York, NY, US: The Guilford Press (2010). 612.

[39] Ortega-Williams A Beltrán R Schultz K Ru-Glo Henderson Z Colón L Teyra C. An integrated historical trauma and posttraumatic growth framework: a cross-cultural exploration. J Trauma Dissociation. (2021) 22:220–40. doi: 10.1080/15299732.2020.1869106, PMID: 33480826

[40] Yehuda R Lehrner A. Intergenerational transmission of trauma effects: putative role of epigenetic mechanisms. World Psychiatry. (2018) 17:243–57. doi: 10.1002/wps.20568, PMID: 30192087 PMC6127768

[41] Tynes BM Willis HA Stewart AM Hamilton MW. Race-related traumatic events online and mental health among adolescents of color. J Adolesc Health. (2019) 65:371–7. doi: 10.1016/j.jadohealth.2019.03.006, PMID: 31196779

[42] Dolezsar CM McGrath JJ Herzig AJM Miller SB. Perceived racial discrimination and hypertension: a comprehensive systematic review. Health Psychol. (2014) 33:20–34. doi: 10.1037/a0033718, PMID: 24417692 PMC5756074

[43] Williams DR Mohammed SA. Discrimination and racial disparities in health: evidence and needed research. J Behav Med. (2009) 32:20–47. doi: 10.1007/s10865-008-9185-0, PMID: 19030981 PMC2821669

[44] Brondolo E Love EE Pencille M Schoenthaler A Ogedegbe G. Racism and hypertension: a review of the empirical evidence and implications for clinical practice. Am J Hypertens. (2011) 24:518–29. doi: 10.1038/ajh.2011.9, PMID: 21331054

[45] Forde AT Sims M Muntner P Lewis T Onwuka A Moore K. Discrimination and hypertension risk among African Americans in the Jackson Heart study. Hypertension. (2020) 76:715–23. doi: 10.1161/HYPERTENSIONAHA.119.14492, PMID: 32605388 PMC8359680

[46] Williams-Washington KN Mills CP. African American historical trauma: creating an inclusive measure. J Multicult Counsel Dev. (2018) 46:246–63. doi: 10.1002/jmcd.12113

[47] Harrington RA Califf RM Balamurugan A Brown N Benjamin RM Braund WE. Call to action: rural health: a presidential advisory from the American Heart Association and American Stroke Association. Circulation. (2020) 141:e615–44. doi: 10.1161/CIR.0000000000000753, PMID: 32078375

[48] Aggarwal R Chiu N Loccoh EC Kazi DS Yeh RW Wadhera RK. Rural-urban disparities. J Am Coll Cardiol. (2021) 77:1480–1. doi: 10.1016/j.jacc.2021.01.032, PMID: 33736831 PMC8210746

[49] Katsinas S. G. Till G. Corley E. G. O’Brien S. Courchesne E. Bray N., Poverty, housing, and GDP in Alabama’s Black Belt. Tuscaloosa, AL: Education Policy Center; The University of Alabama.

[50] Alabama | County health rankings and roadmaps. Available online at: https://www.countyhealthrankings.org/health-data/alabama (accessed: January 30, 2025)

[51] Newman SD Moss K Pichon M Scott D Rogers K Orr A. The health of rural Black communities during COVID: some affirmations, some surprises. Front Public Health. (2023) 11:2451. doi: 10.3389/fpubh.2023.932451, PMID: 37124765 PMC10133505

[52] Kroenke K Spitzer RL Williams JBW Löwe B. An ultra-brief screening scale for anxiety and depression: the PHQ-4. Psychosomatics. (2009) 50:613–21. doi: 10.1176/appi.psy.50.6.613, PMID: 19996233

[53] Oliveros E Patel H Kyung S Fugar S Goldberg A Madan N. Hypertension in older adults: assessment, management, and challenges. Clin Cardiol. (2020) 43:99–107. doi: 10.1002/clc.23303, PMID: 31825114 PMC7021657

[54] Berger T. Places of persistence: slavery and the geography of intergenerational mobility in the United States. Demography. (2018) 55:1547–65. doi: 10.1007/s13524-018-0693-4, PMID: 29971701 PMC6060959

[55] Baker RS. The historical racial regime and racial inequality in poverty in the American south. Am J Sociol. (2022) 127:1721–81. doi: 10.1086/719653

[56] Gaston SA Forde AT Green M Sandler DP Jackson CL. Racial and ethnic discrimination and hypertension by educational attainment among a cohort of US women. JAMA Netw Open. (2023) 6:e2344707. doi: 10.1001/jamanetworkopen.2023.44707, PMID: 37991758 PMC10665977

[57] Forde AT Lewis TT Kershaw KN Bellamy SL Diez Roux AV. Perceived discrimination and hypertension risk among participants in the multi-ethnic study of atherosclerosis. J Am Heart Assoc. (2021) 10:e019541. doi: 10.1161/JAHA.120.019541, PMID: 33596667 PMC8174295

[58] Geronimus AT Hicken M Keene D Bound J. ‘Weathering’ and age patterns of Allostatic load scores among blacks and whites in the United States. Am J Public Health. (2006) 96:826–33. doi: 10.2105/AJPH.2004.060749, PMID: 16380565 PMC1470581

[59] Forde AT Crookes DM Suglia SF Demmer RT. The weathering hypothesis as an explanation for racial disparities in health: a systematic review. Ann Epidemiol. (2019) 33:1–18.e3. doi: 10.1016/j.annepidem.2019.02.011, PMID: 30987864 PMC10676285

[60] Sims M Diez-Roux AV Gebreab SY Brenner A Dubbert P Wyatt S. Perceived discrimination is associated with health behaviours among African-Americans in the Jackson Heart study. J Epidemiol Community Health. (2016) 70:187–94. doi: 10.1136/jech-2015-206390, PMID: 26417003 PMC5014355

[61] Swedo EA Aslam MV Dahlberg LL Niolon PH Guinn AS Simon TR. Prevalence of adverse childhood experiences among U.S. adults — behavioral risk factor surveillance system, 2011–2020. MMWR Morb Mortal Wkly Rep. (2023) 72:707–15. doi: 10.15585/mmwr.mm7226a2, PMID: 37384554 PMC10328489

[62] Su S Wang X Pollock JS Treiber FA Xu X Snieder H. Adverse childhood experiences and blood pressure trajectories from childhood to young adulthood. Circulation. (2015) 131:1674–81. doi: 10.1161/CIRCULATIONAHA.114.013104, PMID: 25858196 PMC4430378

[63] Kreatsoulas C Fleegler EW Kubzansky LD McGorrian CM Subramanian SV. Young adults and adverse childhood events: a potent measure of cardiovascular risk. Am J Med. (2019) 132:605–13. doi: 10.1016/j.amjmed.2018.12.022, PMID: 30639555

[64] Okura Y Urban LH Mahoney DW Jacobsen SJ Rodeheffer RJ. Agreement between self-report questionnaires and medical record data was substantial for diabetes, hypertension, myocardial infarction and stroke but not for heart failure. J Clin Epidemiol. (2004) 57:1096–103. doi: 10.1016/j.jclinepi.2004.04.005, PMID: 15528061

[65] Roberts SJ Chandler GE Kalmakis K. A model for trauma-informed primary care. J Am Assoc Nurse Pract. (2019) 31:139–44. doi: 10.1097/JXX.0000000000000116, PMID: 30550391

[66] Talbot JA Ziller EC Szlosek DA. Mental health first aid in rural communities: appropriateness and outcomes. J Rural Health. (2017) 33:82–91. doi: 10.1111/jrh.12173, PMID: 26817852

[67] Seiling SB Manoogian MM Son S. ‘I Don’t know how we would make it’—social support in rural low-income families, in rural families and work: context and problems In: Bauer JW Dolan EM, editors. International series on consumer science. New York, NY: Springer (2011). 157–83.

[68] Chin D Smith-Clapham AM Wyatt GE. Race-based trauma and post-traumatic growth through identity transformation. Front Psychol. (2023) 14:1602. doi: 10.3389/fpsyg.2023.1031602, PMID: 36844351 PMC9944138

